# Ovarian Cancer-Cell Pericellular Hyaluronan Deposition Negatively Impacts Prognosis of Ovarian Cancer Patients

**DOI:** 10.3390/biomedicines10112944

**Published:** 2022-11-16

**Authors:** Leticia Oliveira-Ferrer, Barbara Schmalfeldt, Johannes Dietl, Catharina Bartmann, Udo Schumacher, Christine Stürken

**Affiliations:** 1 Department of Gynaecology and Gynaecologic Oncology, University Medical Centre Hamburg-Eppendorf, 20246 Hamburg, Germany; 2 Department of Obstetrics and Gynaecology, University of Wuerzburg, 97080 Wuerzburg, Germany; 3 Department of Anatomy and Experimental Morphology, University Medical Centre Hamburg-Eppendorf, 20246 Hamburg, Germany; 4 MSB Medical School of Berlin, 10117 Berlin, Germany; 5 MSH Medical School of Hamburg, University of Applied Sciences and Medical University, 20457 Hamburg, Germany

**Keywords:** ovarian cancer, stromal hyaluronan, tumor-associated hyaluronan staining pattern, hyaluronan-related enzymes

## Abstract

Background: Hyaluronan (HA), a component of the extracellular matrix, is frequently increased under pathological conditions including cancer. Not only stroma cells but also cancer cells themselves synthesize HA, and the interaction of HA with its cognate receptors promotes malignant progression and metastasis. Methods: In the present study, HA deposition in tissue sections was analyzed by hyaluronan-binding protein (HABP) ligand histochemistry in 17 borderline tumors and 102 primary and 20 recurrent ovarian cancer samples. The intensity and, particularly, localization of the HA deposition were recorded: for the localization, the pericellular deposition around the ovarian cancer cells was distinguished from the deposition within the stromal compartment. These histochemical data were correlated with clinical and pathological parameters. Additionally, within a reduced subgroup of ovarian cancer samples (*n* = 70), the RNA levels of several HA-associated genes were correlated with the HA localization and intensity. Results: Both stroma-localized and pericellular tumor-cell-associated HA deposition were observed. Cancer-cell pericellular HA deposition, irrespective of its staining intensity, was significantly associated with malignancy, and in the primary ovarian cancer cohort, it represents an independent unfavorable prognostic marker for overall survival. Furthermore, a significant association between high CD44, HAS2 and HAS3 mRNA levels and a cancer-cell pericellular HA-deposition pattern was noted. In contrast, stromal hyaluronan deposition had no impact on ovarian cancer prognosis. Conclusions: In conclusion, the site of HA deposition is of prognostic value, but the amount deposited is not. The significant association of only peritumoral cancer-cell HA deposition with high CD44 mRNA expression levels suggests a pivotal role of the CD44–HA signaling axis for malignant progression in ovarian cancer.

## 1. Introduction

Ovarian cancer is a heterogeneous, rapidly progressive, highly lethal disease that is poorly understood in its pathophysiology. Due to its lack of specific symptoms, most of the cases are diagnosed at an advanced stage, with little chance of curative treatment; hence, it represents the leading cause of gynecologic cancer deaths in developed countries. The current standard of care for the treatment of the majority of patients with advanced ovarian cancer involves cytoreductive surgery and platinum-based chemotherapy [1]. New therapies with the antiangiogenic agent bevacizumab or poly(adenosine diphosphate-ribose) polymerase (PARP) inhibitors can improve progression-free survival [2,3], but most of the patients ultimately develop recurrent disease that is resistant to chemotherapy [4]. Consequently, it is essential to identify new approaches for treating advanced ovarian cancer.

In addition to targeting the cancer cells directly, a present approach would be to examine the tumor microenvironment in order to detect novel target molecules for ovarian cancer treatment [5]. Malignant progression in most tumor entities is caused not only by mutated signaling networks in the cancer cells themselves but also by important host microenvironmental factors [6,7]. Many studies demonstrate that a host-derived stroma develops concomitantly with primary tumor initiation and therefore provides a nurturing or “cancerized” microenvironment that supports tumor-cell survival, growth and invasion [8,9,10,11].

One such microenvironmental factor is hyaluronan (HA), which is a simple, linear and unsulfated glycosaminoglycan (GAG) composed of repeating disaccharide units of N-acetylglucosamine (GlcNAc) and glucuronic acid (GlcUA). HA is produced at the internal face of the plasma membrane by a family of three hyaluronan synthases named HAS1, HAS2 and HAS3 using cytosolic UDP-GlcNAc and UDP-GlcUA as precursors, and is subsequently extruded to the extracellular space (8–10). These extruded high-molecular-weight HA polymers are first captured by cellular HA receptors (CD44 and RHAMM) [12] and are assembled and stabilized by a family of extracellular HA-binding proteins (such as tenascin, inter-α-trypsin inhibitor, versican and TSG-6) into so-called pericellular matrices [13,14,15]. The synthesis of pericellular HA has the function of organizing and clustering HA receptors on the plasma membrane and sequestering growth factors and cytokines close to the cell membrane [7]. In addition to fibroblasts, metastatic tumor cells may synthesize and assemble their own pericellular HA-rich matrices, thereby maintaining the activation of oncogenic drivers and survival signaling pathways. Thus, HA-rich pericellular matrices facilitate several steps required by circulating tumor cells in the metastatic cascade [7]. Nevertheless, it is worth mentioning that several data compellingly suggest that the autocrine production of pericellular HA matrices alone is not sufficient for successful metastatic colonization. Rather, it appears that tumor cells must also acquire the ability to fragment and metabolize HA to form metastatic colonies [7]. HA can be fragmented by the enzymatic attack of one or more hyaluronidases (e.g., HYAL 1, 2 or 3), resulting in the formation of fragments of different HA sizes [16,17,18].

Aggressive migratory tumor cells that have developed the capacity for autonomous HA synthesis thus incorporate HA into the pericellular matrices and fragment/process the HA polymers to have a selective advantage in metastasis formation [16,19,20].

In the present study, we investigated the amount and deposition pattern of HA located either pericellularly deposited around ovarian cancer cells themselves or deposited within the ovarian cancer stroma. For this purpose, HA deposition was analyzed in a series of borderline tumors and non-invasive ovarian tumors with an excellent prognosis [21,22], as well as in primary and recurrent ovarian cancer samples, and these two parameters were correlated with tumor malignancy and clinical parameters.

## 2. Materials and Methods

### 2.1. Patients

Hyaluronan-binding protein (HABP) staining was analyzed in 17 borderline tumors, and 102 primary ovarian cancer and 20 recurrent ovarian cancer samples. Anonymous archived borderline tumor samples were obtained from the Department of Obstetrics and Gynecology of the University Hospital (Würzburg, Germany). Of the 17 borderline tumors analyzed, 16 were classified as serous or serous-papillary and 1 as mucinous. This information has been added in the revised version of the manuscript. Ovarian cancer samples were obtained during surgery in the University Medical Centre Hamburg-Eppendorf between 2016 and 2020. The ovarian cancer cohort included tissue from different localizations such as ovary, tube and metastatic sites (i.e., omentum majus and parietal peritoneum). The clinical and histological characteristics of the primary carcinomas are shown in Table 1. Survival information was available for 98 primary ovarian cancer patients. Informed consent for the scientific use of tissue materials, which was approved by the local ethics committee (Ethik-Kommission der Ärztekammer Hamburg, #OB/V/03), was obtained from all the ovarian cancer patients. No radiotherapy, neoadjuvant chemotherapy or endocrine therapy had been administered before surgery. The study was performed in accordance with the principles of the Declaration of Helsinki and REMARK criteria [23].

### 2.2. Ligand-Binding Histochemistry Using Hyaluronic Acid-Binding Protein (HABP) and Staining Evaluation

Hyaluronan deposition was detected in tissue sections using a biotinylated hyaluronic acid-binding protein (HABP) as previously described [24]. Briefly, formalin-fixed, paraffin-wax-embedded 4 µm sections were dried overnight at 37 °C, deparaffinized using xylene and rehydrated using a series of graded ethanol solutions. After incubation in a water bath at 60 °C in a 1:10 diluted DAKO retrieval solution (# S1699, Dako, Glostrup, Denmark), the sections were washed in Tris-buffered saline (TBS; 0.05 M Tris-HCl at pH 7.6 and 0.15 M NaCl) and incubated with 1% bovine serum albumin (# K35-001, PAA Laboratories GmbH, Pasching, Austria) in TBS for 30 min. Biotinylated HABP (# 385911 Calbiochem, Merck, Darmstadt, Germany) was diluted 1:75 in Antibody Diluent (# S0809, Dako) and applied to the sections for 1 h at room temperature. Detection of the biotin was performed using the ABC-AP-Kit (# AK-5000, Vector Laboratories Inc., Burlingame, CA), and the Permanent Red Kit (# ZUC001-125, Zytomed Systems, Bargteheide, Germany) was used as a chromogen for the alkaline phosphatase. The sections were counterstained with a hemalum solution (Merck, Darmstadt, Germany), dehydrated and covered with Eukitt (Kindler, Freiburg, Germany) for evaluation.

Two different staining sites were recorded among the ovarian cancers: a pericellular ovarian cancer-cell-associated staining (pericellular staining) and a stromal staining (stromal staining) pattern. The staining intensity score (0: negative, 1: weak, 2: moderate and 3: strong) was recorded for each tissue section independently of the staining site and was evaluated under consideration of the staining intensity and the fraction of positive HA-stained area per sample as previously described [25].

### 2.3. RNA Isolation, Sequencing and RNAseq Analysis

Cryo-cut sections from ovarian cancer samples were stained with hematoxylin and eosin (HE) to assess the quality of the tumor sample. Tissue samples containing more than 70% tumor cells were used for RNA extraction. Next, RNA was harvested by homogenizing the tissue with the QIAshredder and isolating the RNA with the RNeasy Mini kit from QIAgen (Qiagen, Hilden, Germany).

The RNA concentration and RNA quality were assessed using a Bioanalyser (Agilent, Santa Clara, CA, USA) before RNA sequencing was performed by BGI Genomics (Shenzhen, China) using the DNBseq™ Technology Platform in 2 × 100 bp paired-end mode. On average, 24.2 M (minimum: 20.6 M; maximum: 26.1 M) read-pairs were obtained per sample. Data analysis was performed in collaboration with the bioinformatics core facility of the University Medical Center Hamburg-Eppendorf. Sequence reads were aligned to the human reference assembly (GRCh38.95) using STAR (v2.7.0.f) [26], and normalization analysis was carried out with DESeq2 [27].

### 2.4. Statistical Analyses

Statistical analyses were conducted using the SPSS software Version 25 (IBM SPSS Statistics, Armonk, NY, USA). The correlations between the HABP-staining intensity or pattern and tumor malignancy (borderline, primary ovarian cancer and recurrent ovarian cancer), clinicopathologic factors (FIGO, tumor stage, nodal involvement, histological subtype, grading and residual postoperative tumor) and RNA levels were analyzed using chi-square tests. Survival curves were plotted by Kaplan–Meier analysis. Differences between survival curves were evaluated by log-rank tests. Probability values (*p*-value) ≤0.05 were considered statistically significant.

## 3. Results

### 3.1. Hyaluronan Tissue Distribution Pattern but Not Its Intensity Correlates with Tumor Malignancy

**Figure 1:**
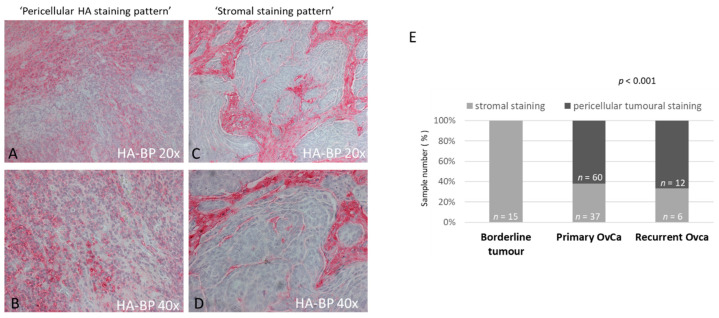
Hyaluronan deposition in ovarian tumors and its association with malignancy. Hyaluronan was detected by ligand histochemistry using a biotinylated hyaluronan-binding protein (HABP) in a patient cohort including 15 borderline tumors, and 102 primary and 20 recurrent ovarian cancer samples. Two staining patterns were detected: fine reticular peri-ovarian cancer-cell HA deposition (**A**,**B**) and coarse stromal HA deposition (**C**,**D**). A significant correlation between fine reticular peri-ovarian cancer-cell HA deposition and malignant potential could be observed (**E**). The *p* value after chi-square tests is provided; *p* < 0.05 was regarded as significant.

Two main types of HA deposition were scored, the first one being a thin pericellular meshwork-like HA-deposition pattern directly around ovarian cancer cells (Figure 1A,B) and the second one represented by a coarser HA-deposition pattern localized in the stroma of the ovarian cancer (Figure 1C,D). Among the borderline tumors, 88% (*n* = 15) showed positive HABP staining (mean value: 1.59), and interestingly, all of them displayed a stromal staining pattern (Figure 1E). In contrast, within the ovarian cancer group, both HA-distribution types were observed. Here, all the primary OvCa presented positive HABP staining (mean value: 1.55), 60 a peritumoral staining pattern, 37 a stromal staining pattern, and 5 samples could not be classified as belonging to any of these two groups, as they showed a mixture of both staining patterns and were therefore designated as mixed phenotype. Within the subgroup of 20 recurrent ovarian cancers, 19 samples (95%) were positive for HABP staining (mean value: 1.40), and among them, 12, 6 and 1 were classified as peritumoral, stromal and mixed phenotype, respectively. Statistical analysis (ANOVA) confirmed a significant difference in the staining pattern between borderline tumors and carcinomas—both primary and recurrent (*p* < 0.001)—but not between primary and recurrent tumors.

### 3.2. Peritumorally Associated Hyaluronan Deposition Is Associated with Shorter Overall Survival in Ovarian Cancer Patients

In the subgroup of primary ovarian carcinomas (*n* = 102), the HABP-staining pattern was also correlated with histopathological and clinical parameters.

**Figure 2:**
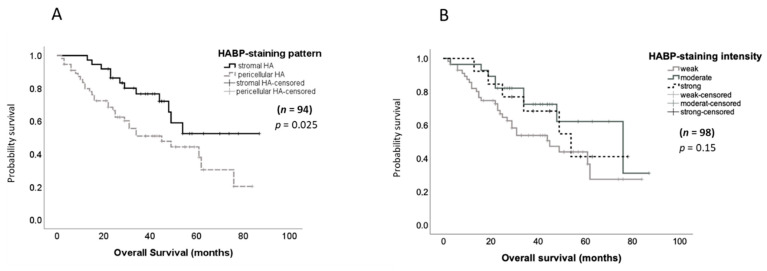
Kaplan–Meier analysis of overall survival based on HABP-staining intensity and staining pattern. (**A**) Kaplan–Meier analysis showed a significant correlation of pericellular ovarian cancer-cell HA deposition with shorter overall survival. (**B**) No significant association of HA-deposition intensity with overall survival including all primary tumors could be found. *p* values after log-rank tests are shown; *p* < 0.05 was regarded as significant.

The HABP-staining pattern significantly correlated with patient overall survival. Here, patients displaying tumor-associated HABP staining showed significantly shorter survival rates in comparison to those patients with a stromal HA pattern (*p* = 0.025), as displayed in the Kaplan–Meier curve (log-rank) in Figure 2A. In multivariate Cox regression analysis including the most important prognostic parameters for ovarian cancer, the residual tumor after surgery and FIGO stage, the HABP-staining pattern turned out to be an independent prognostic parameter for shorter survival for this tumor entity (*p* = 0.035; hazard ratio = 2.297).

In contrast, no significant associations (chi-square test) were found between the HA-deposition pattern and FIGO classification, clinical stage, nodal involvement, distant metastasis and residual tumor after surgery (Supplementary Materials; Figure S1).

### 3.3. High Stromal Hyaluronan Expression Showed No Impact on Survival of Ovarian Cancer Patients

To assess the potential impact of HA deposition on cancer progression, the correlations between the HABP-staining intensity and histopathological and clinical parameters were investigated in the subgroup of primary ovarian carcinomas (*n* = 102). The staining intensity (0: negative, 1: weak, 2: moderate and 3: strong) was scored for each tissue section, independently of the staining pattern. According to Kaplan–Meier analysis and log-rank tests, no association of the HABP-staining intensity with disease-free (*p* = 0.928, data not shown) or overall survival (OAS) (*p* = 0.153, Figure 2B) could be observed. Furthermore, chi-square tests revealed no significant associations of HABP staining with any of the analyzed parameters, such as FIGO, clinical stage, nodal involvement, distant metastasis and residual tumor after surgery (Supplementary Materials; Figure S2). A trend towards higher expression in high-grade ovarian cancer samples was noticed (*p* = 0.082). 

### 3.4. Tumor-Associated Hyaluronan Expression Is Associated with Higher HA-Synthase and CD44 mRNA Levels

**Figure 3:**
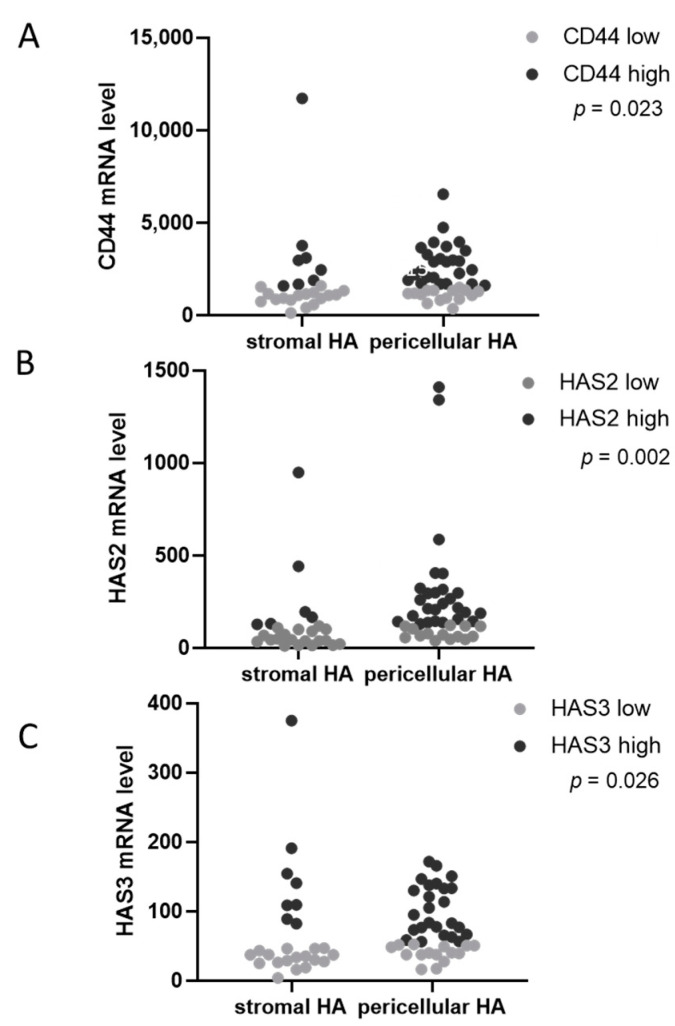
Correlations between HA-deposition pattern and expression of HA-related genes. Tumors displaying an ovarian cancer-cell pericellular hyaluronan deposition pattern showed higher mRNA levels of (**A**) CD44, (**B**) HAS2 and (**C**) HAS3. *p* values after chi-square tests are provided; *p* < 0.05 was regarded as significant.

From a subgroup of primary ovarian cancer samples (*n* = 70), RNAseq data were available. We subsequently examined whether the mRNA levels of HA-related genes were associated with the HABP-staining intensity or staining pattern observed in the ligand HABP histochemistry. The mRNA levels of three hyaluronan synthases (HAS1, HAS2 and HAS3), three hyaluronidases (HYAL1, HYAL2 and HYASL3) and the main receptors CD44 and RHAMM were evaluated. For each factor, the cohort was divided into two groups with low or high mRNA levels with the median as the cut-off value. By chi-square tests, these groups were statistically compared in terms of the HABP-staining intensity and pattern. No significant association of the HABP intensity with any of the analyzed parameters was detected. However, a significant correlation between high CD44, HAS2 and HAS3 mRNA levels and pericellular hyaluronan tumor staining, as shown in Figure 3A–C, respectively, was found.

## 4. Discussion

In the present work, we describe the strong impact of the HA localization, rather than its quantity, on malignant progression and thus on the prognosis of ovarian cancer patients.

In our cohort, including borderline tumors and primary and recurrent ovarian cancer samples, we observed two main HA-deposition patterns by ligand histochemistry using a biotinylated HABP, the first one being localized directly around ovarian cancer cells and the second one present in the connective tissue stroma distant from the cancer cells. The latter finding has been reported before for several tumor entities, including ovarian cancer [28,29,30,31]. Here, stromal HA expression has previously been described as an unfavorable prognostic factor in breast, gastric or colorectal cancer [26,29,30,31]. However, much less attention has been paid to the pericellular deposition of HA around the ovarian cancer cells themselves. Interestingly, borderline tumors exclusively displayed stromal hyaluronan, whereas within the ovarian cancer cohort, both stromal and cancer-cell-associated HA was found, indicating an association of intratumoral hyaluronan with increased malignancy. Indeed, in the ovarian cancer cohort, the HA-distribution pattern, independently of the deposition intensity, showed a clear impact on the prognosis of ovarian cancer patients. Here, patients displaying tumor ovarian cancer-cell-associated HA showed a significantly shorter overall survival than those with a stromal HA distribution. In line with the data from Anttila et al. [28], tumor-cell-associated HA did not correlate with FIGO, the clinical stage or recurrence, and only a trend towards poorer tumor differentiation was observed. Stromal HA expression has previously been described as an unfavorable prognostic factor in breast, gastric or colorectal cancer [29,32,33,34].

Numerous in vitro and in vivo studies have demonstrated the relevance of hyaluronan production by tumor cells themselves for their malignant behavior, including increased tumor-cell proliferation and migration, apoptosis prevention and EMT induction in diverse tumor entities (e.g., colon and gastric cancers) [35]. Here, the overexpression of hyaluronan synthases (HASes) increased local tumor growth [36,37] or distant metastasis [38,39,40], whereas HA-synthesis inhibition reduced tumor progression [41] and spreading [42] in in vitro breast, prostate and colon cancer-cell models. These data are in line with our results, as they underline the key role of tumor-associated HA secretion and membrane-dependent binding for malignant progression. HA exerts its biological functions through two different receptors, CD44 and RHAMM. CD44 expression at the tumor-cell surface has been shown to interact with hyaluronan in the microenvironment, thereby activating signaling pathways that induce migration, invasion and metastasis [43], while RHAMM acts on the intracellular HA concentration. In the present study, and in line with these data, higher CD44 mRNA levels were detected in those samples displaying ovarian cancer-cell-associated HA deposition compared to those with a stromal HA pattern. Here, concomitant CD44 expression and HA pericellular deposition on ovarian cancer cells might result in pro-tumorigenic signaling activation, thereby explaining the more malignant tumor phenotype and the poorer prognosis of those patients displaying an ovarian cancer-cell-associated HA-deposition pattern.

Contrary to previously published results [28,44], the HA-deposition intensity was not prognostic in our ovarian cancer cohort. Here, a different evaluation procedure for the HA staining might explain this discordance. In the mentioned study, merely the intensity of the HA stromal staining was considered for survival analysis, whereas in the present work, both ovarian cancer-cell-associated and stromal HA expression was distinguished.

## 5. Conclusions

Taken together, the present study reveals ovarian cancer-cell-associated hyaluronan deposition as an independent prognostic parameter in ovarian cancer. The significant association of tumoral HA with high CD44 levels suggests a pivotal role of this signaling axis for progression for this entity.

## Figures and Tables

**Table 1:** The clinical and histological characteristics of the primary carcinomas.

Cohort Description		Patient Number
Tumor Classification (*n* = 139)	Borderline tumor	17
	Recurrent ovarian cancer	20
	Primary ovarian cancer	102 *
Age at diagnosis (y)	mean (median)	59.3 y (60 y)
Histological Type	serous-papillary	93
	endometrioid	2
	clear cell	1
	muelerian mucinous	5
FIGO stage	FIGO I-II	2
	FIGO IIIA-IIIB	6
	FIGO IIIC	69
	FIGO IV	17
Nodal involvement	negative	14
	positive	54
Grading	lg	3
	hg	85
Distant metastasis	negative	72
	positive	20
Tumor residuum after surgery	not macroscopically visible	53
	<1 cm^3^	23
	>1 cm^3^	25
Adj. Chemotherapy	Carboplatin/Paclitaxel (Taxol)	97
	Bevacizumab (maintenance)	61
Recurrence	yes	70
	no	32
Recurrence-free survival (months)	mean (median)	23.88 (21)
Overall survival (months)	mean (median)	36 (33,5)
Follow-up (months)	mean (median)	45.2 (39)

*: missing values to *n* = 102: unknown.

## Data Availability

The data that support the findings of this study are available upon request to the corresponding author with appropriate institutional approvals. The data are not publicly available due to privacy and ethical restrictions.

## Supplementary Materials

The following supporting information can be downloaded at: https://www.mdpi.com/article/10.3390/biomedicines10112944/s1, Figure S1: Correlations between HA staining pattern and histopathologic parameter; Figure S2: Correlations between HA staining intensity and histopathologic parameter.

## Abbreviations

HA	Hyaluronan
HABP	Hyaluronan-binding protein
HAS	Hyaluronan synthase
HYAL	Hyaluronidase
PARP	Poly(adenosine diphosphate-ribose) polymerase
GAG	Glycosaminoglycan
GlcNAc	Acetylglucosamine
GlcUA	Glucuronic acid
RHAMM	Receptor for HA mediated motility
