# Supplementary material for: Ovarian Cancer-Cell Pericellular Hyaluronan Deposition Negatively Impacts Prognosis of Ovarian Cancer Patients
Source: Biomedicines. 2022 Nov 16;10(11):2944. doi: 10.3390/biomedicines10112944 (PMC9687866; doi:10.3390/biomedicines10112944)
Supplement: Supplementary file 1 [file biomedicines-10-02944-s001.zip › biomedicines-1947109-supplementary.pdf]

Supplementary Material

Figure S1: Correlations between HA staining pattern and histopathologic parameter

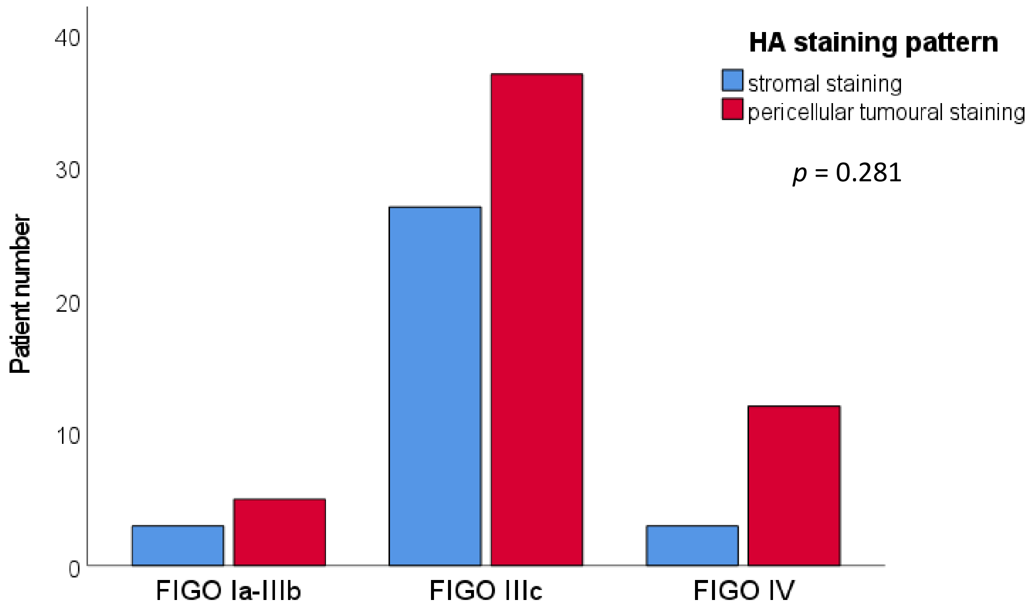

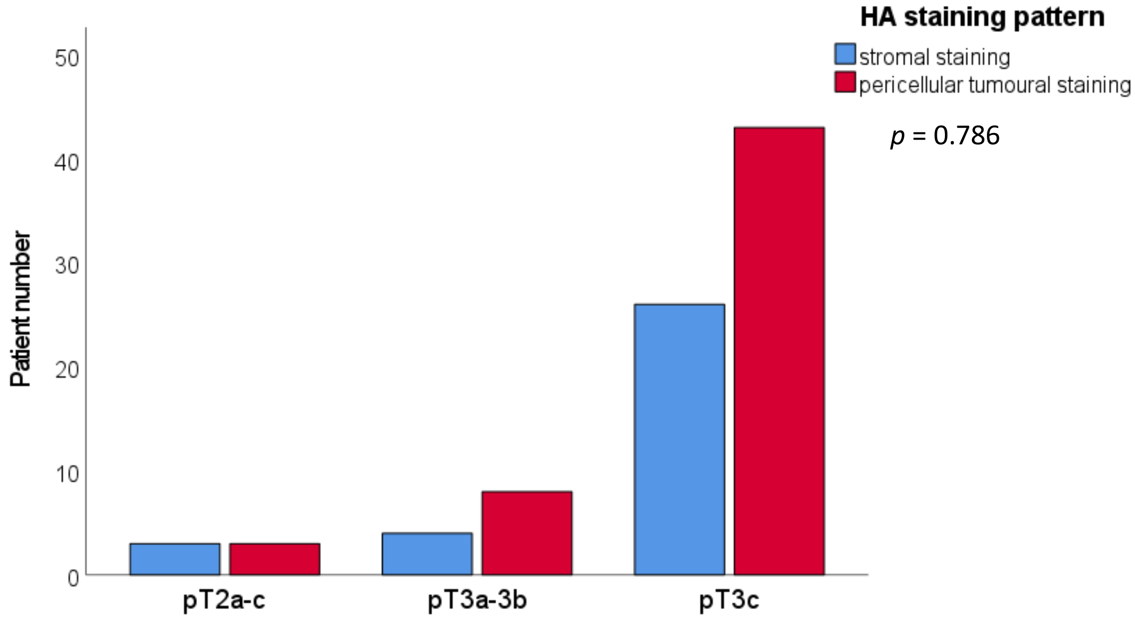

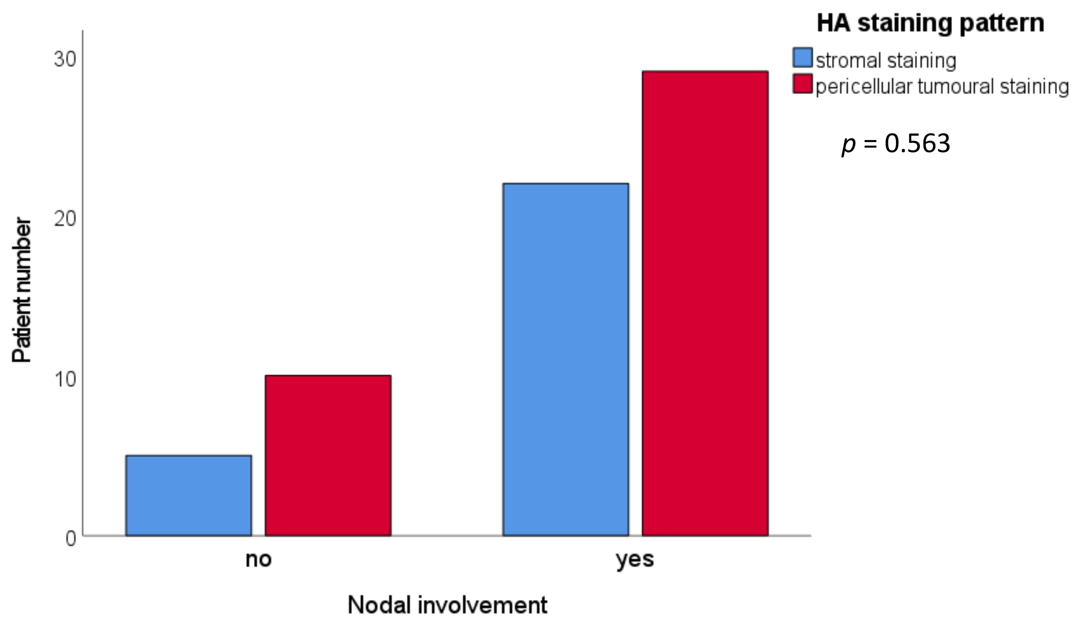
HA staining pattern
stromal staining
pericellular tumoural staining
p = 0.563
Patient number
0
10
20
30
no
yes
Nodal involvement

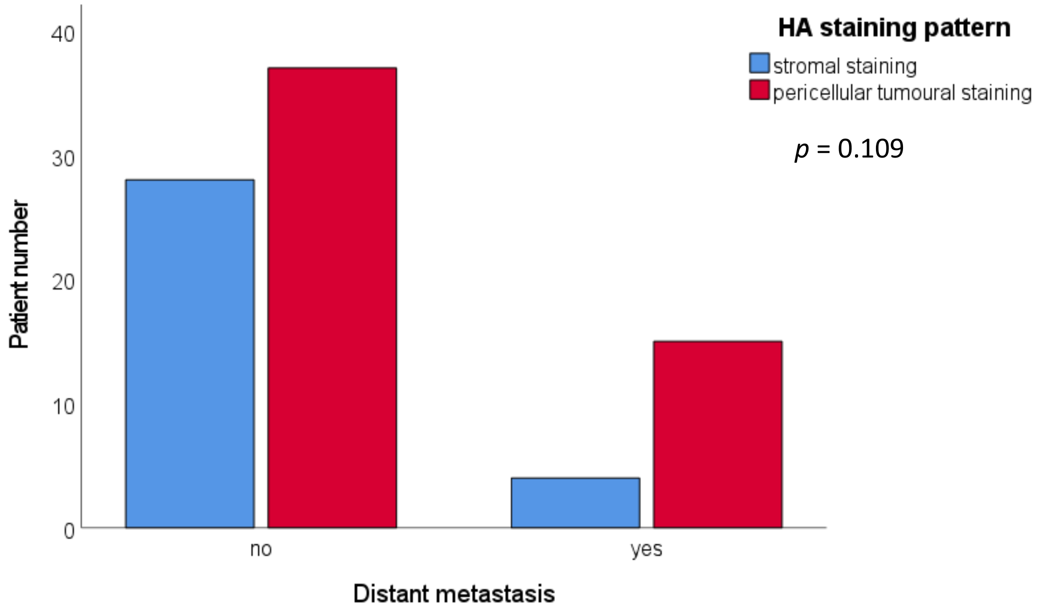
HA staining pattern
stromal staining
pericellular tumoural staining
p = 0.109
Patient number
0
10
20
30
40
no
yes
Distant metastasis

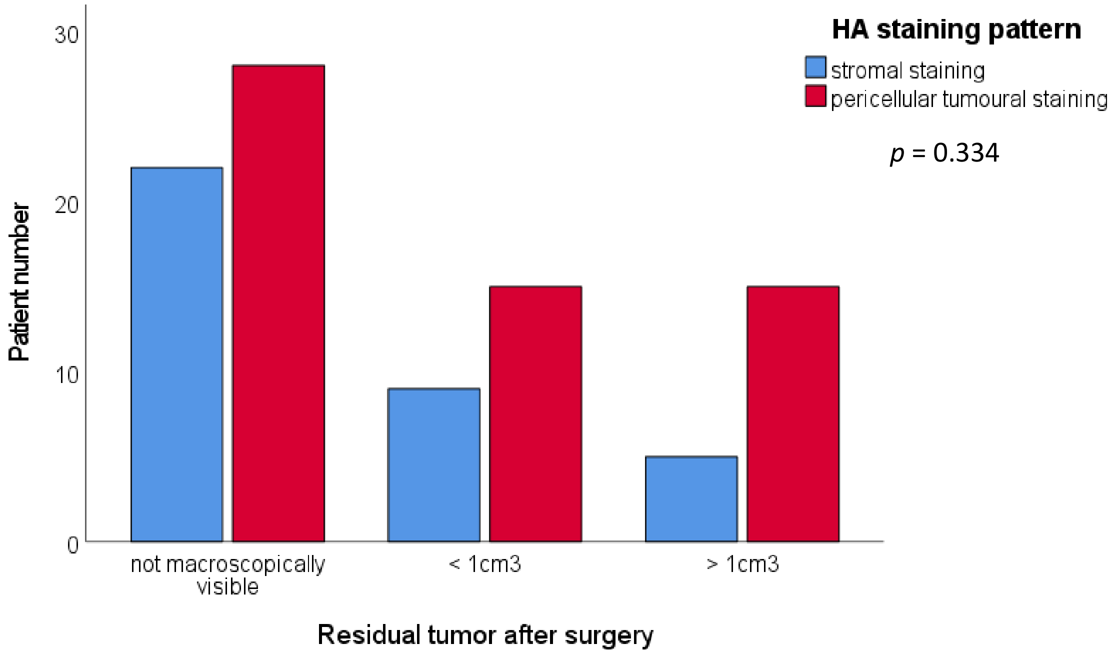
HA staining pattern
stromal staining
pericellular tumoural staining
p = 0.334
Patient number
0
10
20
30
not macroscopically visible
< 1cm3
> 1cm3
Residual tumor after surgery

Figure S2: Correlations between HA staining intensity and histopathologic parameter

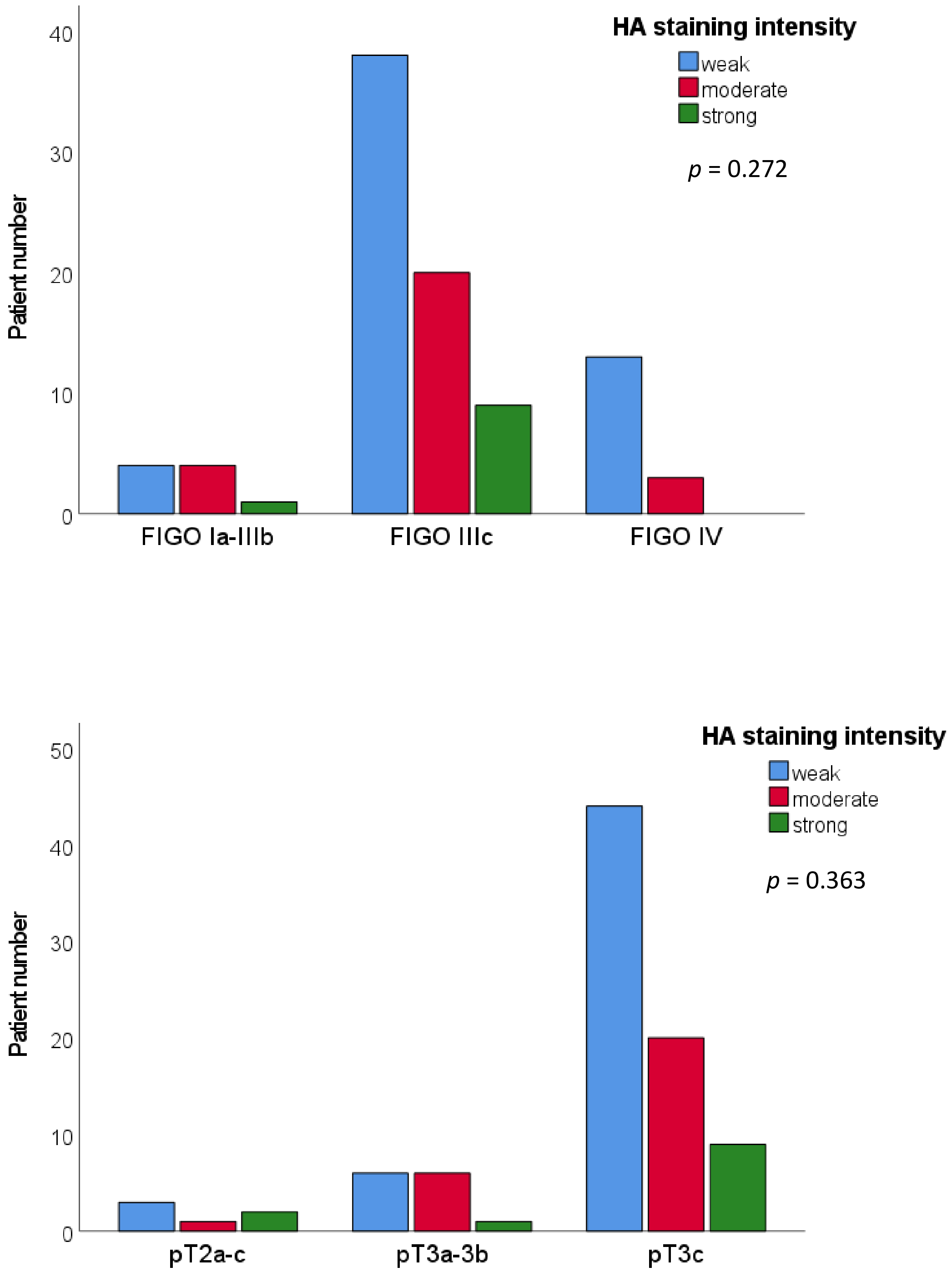

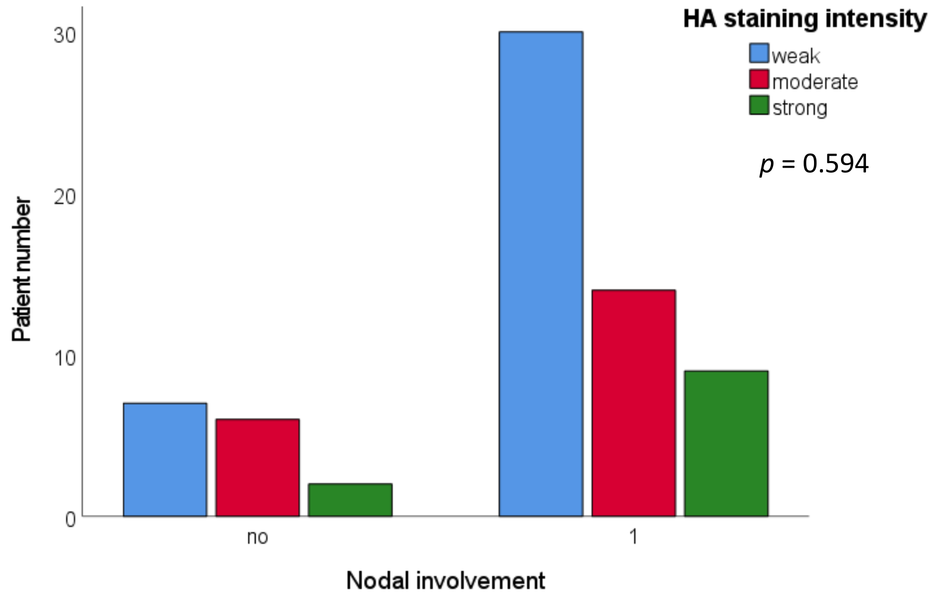
HA staining intensity
weak
moderate
strong
p = 0.594
Patient number
30
20
10
0
no
1
Nodal involvement

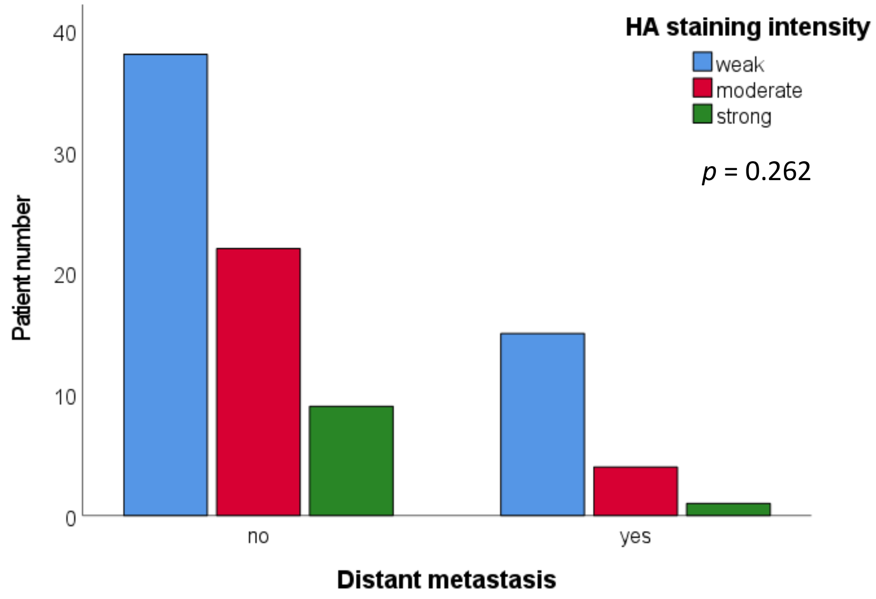
HA staining intensity
weak
moderate
strong
p = 0.262
Patient number
40
30
20
10
0
no
yes
Distant metastasis

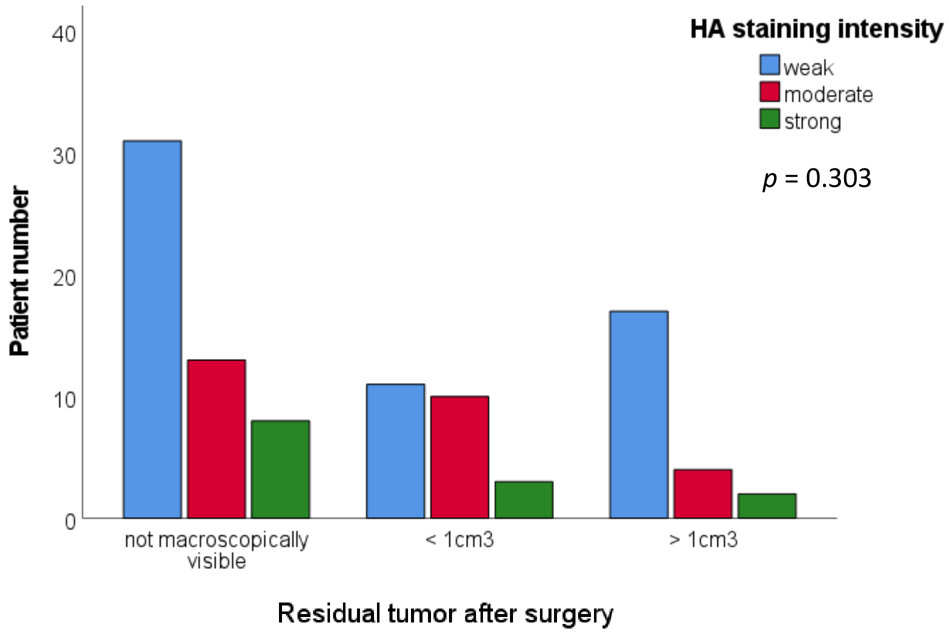
HA staining intensity
weak
moderate
strong
p = 0.303
Patient number
40
30
20
10
0
not macroscopically visible
< 1cm3
> 1cm3
Residual tumor after surgery
